# A practical guide for the husbandry of cave and surface invertebrates as the first step in establishing new model organisms

**DOI:** 10.1371/journal.pone.0300962

**Published:** 2024-04-04

**Authors:** Marko Lukić, Lada Jovović, Jana Bedek, Magdalena Grgić, Nikolina Kuharić, Tin Rožman, Iva Čupić, Bob Weck, Daniel Fong, Helena Bilandžija

**Affiliations:** 1 Department of Molecular Biology, Ruđer Bošković Institute, Zagreb, Croatia; 2 Croatian Natural History Museum, Zagreb, Croatia; 3 Croatian Biospeleological Society, Zagreb, Croatia; 4 Department of Biology, Southwestern Illinois College, Belleville, Illinois, United States of America; 5 Department of Biology, American University, Washington, DC, United States of America; CNRS UMR9197, FRANCE

## Abstract

While extensive research on traditional model species has significantly advanced the biological sciences, the ongoing search for new model organisms is essential to tackle contemporary challenges such as human diseases or climate change, and fundamental phenomena including adaptation or speciation. Recent methodological advances such as next-generation sequencing, gene editing, and imaging are widely applicable and have simplified the selection of species with specific traits from the wild. However, a critical milestone in this endeavor remains the successful cultivation of selected species. A historically overlooked but increasingly recognized group of non-model organisms are cave dwellers. These unique animals offer invaluable insights into the genetic basis of human diseases like eye degeneration, metabolic and neurological disorders, and basic evolutionary principles and the origin of adaptive phenotypes. However, to take advantage of the beneficial traits of cave-dwelling animals, laboratory cultures must be established—a practice that remains extremely rare except for the cavefish *Astyanax mexicanus*. For most cave-dwelling organisms, there are no published culturing protocols. In this study, we present the results of our multi-year effort to establish laboratory cultures for a variety of invertebrate groups. We have developed comprehensive protocols for housing, feeding, and husbandry of cave dwellers and their surface relatives. Our recommendations are versatile and can be applied to a wide range of species. Hopefully our efforts will facilitate the establishment of new laboratory animal facilities for cave-dwelling organisms and encourage their greater use in experimental biology.

## Introduction

Life sciences disciplines necessitate work with living organisms, particularly in evolutionary and developmental biology, behavioral ecology, and genetics, among many others [1–3]. Rapid advances in these disciplines were made possible with the use of model organisms such as laboratory mice, zebrafish, fruit flies, and nematodes. Although standardized protocols to breed and rear these model organisms are widely available, using live animals is a challenging task and laboratory care for even well-established model organisms can have unpredictable outcomes [e.g. 4].

The nature of much research, however, necessitates use of non-model organisms, and establishment of a thriving and breeding laboratory colony is vital and a major hurdle. A laboratory stock of experimental organisms has two obvious advantages. First, it guarantees continuous availability of specimens with desired characteristics enabling standardization and replicability [5–8]. Second, having a laboratory stock reduces the need for repeated removal of specimens from natural populations. This has positive implications on the protection and conservation of species with low population sizes or threatened or endangered status, often the original impetus to study these species [9–11]. Yet, protocols for the husbandry and successful breeding of non-model organisms are rare, because of the difficulties in establishing optimal conditions in the laboratory, and because triggers for reproduction are usually unknown. The need for and the obstacles in establishing laboratory colonies of non-model organisms are especially acute in species that live in underground ecosystems [12].

Cave-dwelling species are emergent models useful for addressing some of the fundamental questions in biology, such as how organisms adapt to new and extreme environments, how novel phenotypes evolved and what are the mechanism underlying one of the most fascinating examples of convergence in nature [13–15]. This is because cave species: i) can bear the same suite of traits, including the loss of eyes and pigmentation, changes in metabolism, increases in appendage length and non-visual sensory systems, that arose independently across different phyla in the animal kingdom, ii) caves are ecologically simple and the environmental cues (darkness and nutrient depletion) that correlate with distinct cave adapted phenotypes are well defined, and iii) the ancestral form (surface relatives) is sometimes available for comparative studies. Further, a suite of characteristics of cave animals such as albinism, eye degeneration, extended longevity, metabolic and immune changes, sleep loss and neurological alterations offer great potential for biomedical research because some of these characteristics are reminiscent of symptoms of diseases in humans. Understanding the genetic bases, physiological consequences, and environmental contributions of these adaptations is of particular interest because it may help discover rare genetic variants, environmental predictors, and alternative homeostatic states. Therefore, cave animals may serve as models to study the molecular basis and potential treatments of such diseases, including diabetes, stress, metabolism dysfunction, sleep disorders, and aging [16–25]. While studies like this have focused only on a few cave vertebrate species, most notably the Mexican cavefish *Astyanax mexicanus* and to a less extent salamanders such as the olm *Proteus anguinus*, cave invertebrates offer great potential for studies of the universality of genetic mechanisms underlying these characteristics. Invertebrates are ubiquitous and far more taxonomically diverse than vertebrates in caves and offer many cases of multiple independent evolution of superficially similar phenotypes. However, there is a large gap in culturing cave invertebrates compared to cave vertebrates.

This study stems from the lack of published protocols and difficulty of maintaining subterranean species outside their natural environment [12]. Successful examples of breeding cave fauna in captivity come from a few subterranean laboratories located in natural caves, like Moulis Cave (France), Bossea Cave (Italy) and Tular Cave Laboratory (Slovenia) [26–28]. Despite these successes, culturing protocols are usually poorly described and are scattered in relatively inaccessible journals [29–31]. In subterranean ecosystems, complete darkness, buffered climatic conditions, and food scarcity are key ecological features that cannot be easily replicated in the laboratory. Although darkness can be mimicked in the laboratory, complete darkness cannot be maintained because of the need for researchers to conduct experiments, and the effects of occasional exposure of cave animals to light is not well understood. Subterranean species have adapted to stable environment conditions such as constant temperature and relatively high humidity, and seem unable to cope with fluctuations in environmental conditions [32–34]. Therefore, even small changes in environmental conditions in the laboratory may have detrimental effects [32, 35–37]. Food scarcity may be mimicked by withholding food in the laboratory, but what they feed on in the wild and how often are unclear. Finally, due to the cryptic habitat and lifestyle of cave animals, little is known about their life history (for example [30, 38–46]) further complicating their propagation in the laboratory.

**Fig 1:**
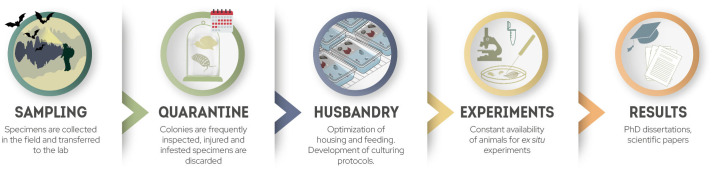
Schematic diagram of steps necessary to establish a culture of wild species to be used in laboratory experiments. Credit: Iva Čupić, Tin Rožman.

In this article, we summarize our experience in setting up invertebrate laboratory facilities to culture and study cave animals. We provide recommendations for maintaining taxonomically related invertebrates from subterranean and surface habitats in laboratory cultures (Fig 1). These guidelines are the result of a three-year effort to test and refine cultivation protocols for new non-model organisms, with staff dedicated solely to this purpose. The data presented here are useful for establishing invertebrate laboratory facilities in general and can also be applied to a broader list of species from various taxonomic groups.

## Materials and methods

### Species

Species used in this study were selected according to the following criteria. 1. Close phylogenetic relationship between cave-adapted and surface population. If surface populations of the cave species do not exist, as closely related as possible surface species or genus was chosen. 2. Population abundance high enough to ensure sufficient individuals could be collected to start the laboratory colony without endangering the population. 3. Ease of access to the habitat and the simplicity of sampling. Specimens may not survive the physical buffeting when exiting technically demanding caves. 4. Ability to identify the species *in-situ*, i.e., species without close relatives in the same locality.

**Fig 2:**
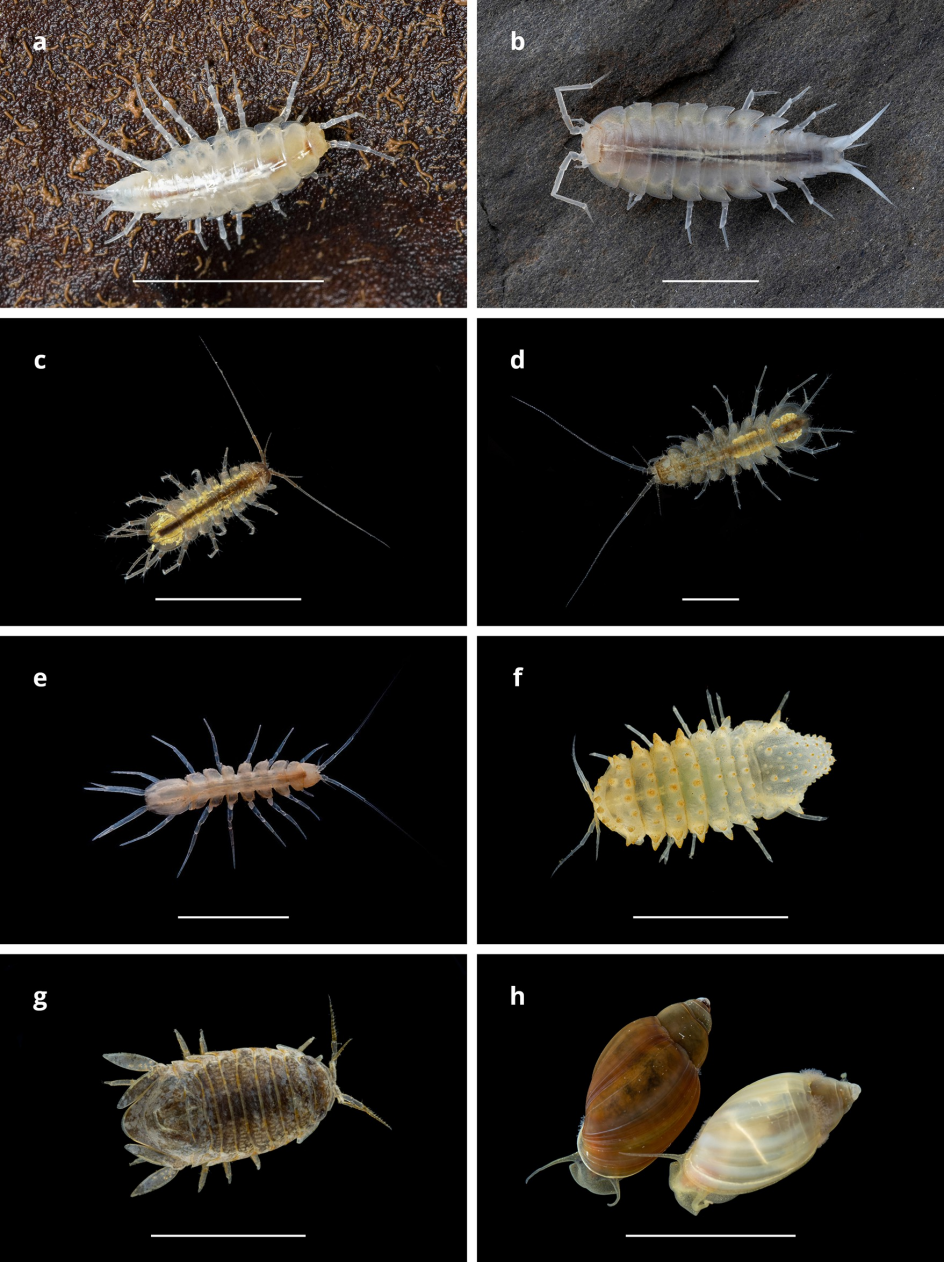
Photos of selected animals used in our studies, one species per genus. A. *Alpioniscus balthasari*. B. *Titanethes albus*. C. *Proasellus karamani*. D. *Asellus aquaticus*. E. *Ceacidotea pricei*. F *Monolistra pretneri*. G. *Lekanesphaera hookeri*. H. *Physella* sp. Scale bars, 5 mm. Credits: Jana Bedek (a), Mike Slay (e), Tin Rožman (b, c, d, f, g, h).

A complete list of species used in this study is given in S1 Table. Only information on the species we successfully cultivated (Tables 1 and 2, Fig 2) is detailed below. Among crustaceans we established colonies of isopod species from three different families: 1) aquatic Asellidae (surface: *Proasellus coxalis* s.l., *P*. *karamani*, *Asellus aquaticus*, and *Caecidotea kenki*; cave: *P*. *anophtalmus*, *P*. *hercegovinensis*, *A*. *aquaticus*, and *C*. *pricei*), 2) aquatic Sphaeromatidae (surface: marine *Lekanesphaera hookeri*; cave: freshwater *Monolistra pretneri* and *M*. *velkovrhi*), and 3) terrestrial Trichoniscidae (cave: *Alpioniscus balthasari* and *Titanethes albus*) (Fig 2a–2g). In addition, a colony of a cave population of the gastropod *Physella* sp. was established (Fig 2h). Details on the habitat and distribution of these species are given in S1 Appendix.

**Table 1:** Summary table for the culturing conditions and maintenance requirements for cave and surface invertebrates that were successfully maintained in laboratory for this study.

	Species	Habitat	No. of collecting events *	Housing	Feeding frequency (conditioned leaves / food pellets)	Care frequency	Hands on time **
CRUSTACEA	Trichoniscidae						
	*Alpioniscus balthasari* (Frankenberger, 1937)	terrestrial / cave	<5	200 mL containers with moistened plaster	*ad libitum / –*	once a month	2
	*Titanethes albus* (C. Koch, 1841)	terrestrial / cave	<5	1 L containers with moistened plaster, stones and large water pools	*ad libitum / –*	every two weeks	4
	Asellidae						
	*Caecidotea kenki* (Bowman, 1967)	aquatic / seepage spring	<5	3 L containers	*ad libitum / –*	twice per week	5
	*Caecidotea pricei* (Levi, 1949)	aquatic / cave	1	3 L containers	*ad libitum / fish flakes once a month*	twice per week	5
	*Asellus aquaticus* (Linnaeus, 1758)	aquatic / surface and cave	>5	1–3 L containers	*ad libitum /* once a month	once a month	5
	*Proasellus coxalis* (Dollfus, 1892) s.l.	aquatic / surface	>5	1–3 L containers	*ad libitum /* once a month	once a month	5
	*Proasellus karamani* (Remy, 1934)	aquatic / surface	<5	1–3 L containers	*ad libitum /* once a month	once a month	5
	*Proasellus anophtalmus* (Karaman, 1934)	aquatic / cave	<5	1–3 L containers	*ad libitum /* once a month	once a month	5
	*Proasellus hercegovinensis* (Karaman, 1933)	aquatic / cave	<5	1–3 L containers	*ad libitum /* once a month	once a month	5
	Sphaeromatidae						
	*Monolistra pretneri* Sket, 1964	aquatic / cave	<5	1 L containers	*ad libitum / –*	every two weeks	10
	*Monolistra velkovrhi* Sket, 1960	aquatic / cave	<5	1 L containers	*ad libitum / –*	every two weeks	10
	*Monolistra radjai* Prevorčnik & Sket, 2007	aquatic / cave	<5	1 L containers	*ad libitum / –*	every two weeks	10
	*Lekanesphaera hookeri* (Leach, 1814)	marine / estuary	<5	1 L containers	*ad libitum /* once a month	every week	20
GASTROPODA	Physidae						
	*Physella* sp.	aquatic / cave	1	200 ml, 1 L, 3.8 L containers	– / 2-3- times a week, once per week in 3.8L container	2–3 times a week, once per 2 weeks in 3.8 L container	32; 20 hours for 3.8 L containers

* No. of collecting events refers to the number of times organisms were collected from the wild to establish the lab colony (as 1, <5 or >5)

** Hands on time needed to maintain the laboratory cultures is given in hours per month for 100 containers.

**Table 2:** Summary table of cave and surface invertebrates that were successfully maintained in laboratory for this study. We included species that were either breeding or had high survival as adults in laboratory environment. A complete list of all species and sampling sites used in this study with comments on survival and culturing success per site is given in S1 Table. Scores low (L), medium (M), and high (H) for survival of the wild individuals in the lab and for survival of offspring denotes <20%, 20–80% and >80% of individuals surviving, respectively. Frequency of reproduction is defined as >1 or <1 per year.

Group/species	Survival of the wild individuals in the lab	Frequency of reproduction	Survival of offspring	Generation time	No. of generation in this study **
Trichoniscidae					
*Alpioniscus balthasari* (Frankenberger, 1937)	H	N/A	N/A	unknown	N/A
*Titanethes albus* (C. Koch, 1841)	M	N/A	N/A	unknown	N/A
Asellidae					
*Caecidotea kenki* (Bowman, 1967)	H	>1	M	1 year	1–2
*Caecidotea pricei* (Levi, 1949)	M	<1	L	1–2 years	1
*Asellus aquaticus* (Linnaeus, 1758), surface	H	>1	M	1 year	3
*Asellus aquaticus* (Linnaeus, 1758), cavelike*	M/H	<1	L/M	1–2 year	2
*Proasellus coxalis* (Dollfus, 1892) *s*.*l*	H	>1	M	6 months	4
*Proasellus karamani* (Remy, 1934)	H	>1	M	unknown	2
*Proasellus anophtalmus* (Karaman, 1934)	H	<1	M	unknown	N/A
*Proasellus hercegovinensis* (Karaman, 1933)	H	<1	L	unknown	1
Sphaeromatidae					
*Monolistra pretneri* (Sket, 1964)	M	N/A	N/A	unknown	N/A
*Monolistra velkovrhi* (Sket, 1960)	M	N/A	N/A	unknown	N/A
*Lekanesphaera hookeri* (Leach, 1814)	M	<1	L	unknown	N/A
Physidae					
*Physella* sp.	H	>1	M	Approx. 70 days	plur.

* cavelike population refers to not fully troglomorphic populations of *Asellus aquaticus* from caves Lummelundagrottan (Sweden) and Sušik ponor (Croatia)

** number of generations in this study is given at the time when manuscript was submitted, but this is not the definite number of laboratory generations as the colonies are still in the lab and reproducing (except for *Caecidotea kenki* and *C*. *pricei*).

All experiments were performed in accordance with relevant institutional and national guidelines for the care and use of laboratory animals. Species in the wild were collected under the following permits: UP/I-612-07/20-48/83, 517-05-1-1-20-5; UP/I-352-04/22-08/96, 517-10-1-1-22-5; 521-2215-2021; 35602-41/2021-5; NH226599; Nature Preserves Commission Permit # 2020 INPC ROBERT WECK FOGELPOLE CAVE; GWMP-2015-SCI-0006 (Dan Fong, Pimmit Run Seepage Spring, *Caecidotea kenki*); VADNR-DNH open permit (Wil Orndorff, Ogden Cave, *Caecidotea pricei*).

### Sampling and quarantine

The wild animals were collected using tweezers, brushes, transfer pipettes, large pipettes (turkey baster), nets, and aspirators. Individuals of both sexes and random ages were collected, up to 30 specimens per sampling location and/or event for protected cave-dwelling species and multiple sampling events were carried out over the course of the years (Table 1). Basic physical parameters of the sampling site such as water, air or soil temperature, pH, electrical conductivity, salinity, and oxygen percentage were recorded. Samples were transported to the laboratory in styrofoam boxes with ice packs or in an electric car cooler. All animals were first quarantined for 14 days in a dedicated space under appropriate conditions. Aquatic animals were kept in their native water, which was gradually replaced with facility water during regular water changes. After quarantine, animals that were injured, malformed or infested with parasites were discarded and the rest were relocated to the culturing facility and were registered in an entry form. New colonies were inspected at least once a week for signs of stress and disease.

### Housing

**Fig 3:**
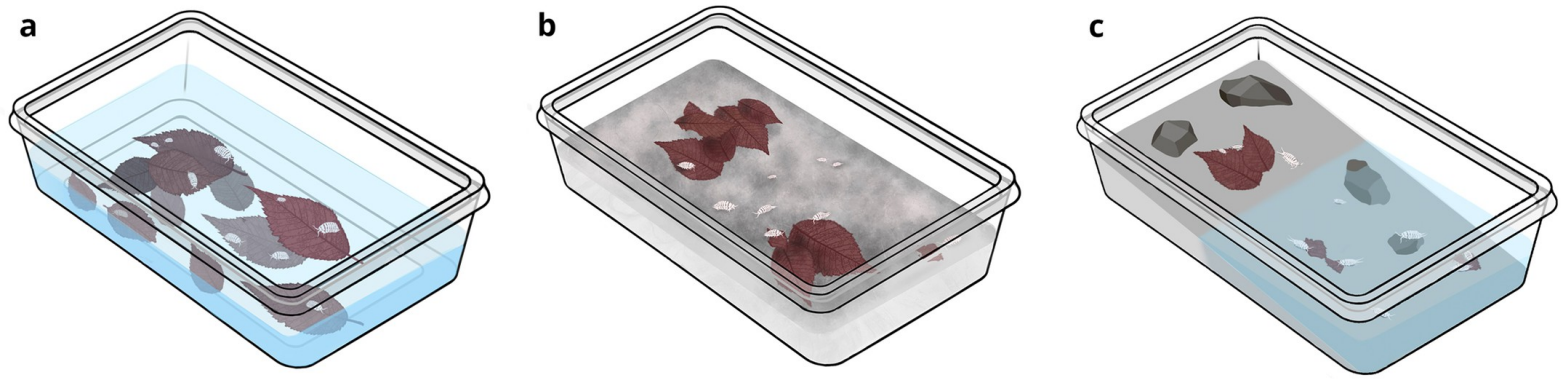
Representation of housing used in our studies. A. Plastic containers used for aquatic animals. B. Plastic containers with bottom made of plaster used for terrestrial animals. C. Plastic containers with a plaster bottom inclined to form a large pool of water optimal for rearing larger semi-aquatic cave trichoniscids. Credit: Iva Čupić.

Animals were kept in low profile plastic containers (polypropylene–PP (5)) ranging from 30 mL to 3 L depending on the species and population size (Table 1). Aquatic species were kept in loosely lidded containers with water up to approximately one third for crustaceans and up to two thirds of the container height for physid snails (Fig 3a), resulting in a high surface area to volume ratio to allow gas exchange without the need for aeration. Aquarium air filters or additional aeration were not used because they increased the rate of microbial contamination in preliminary setups.

Terrestrial species were kept in containers of various sizes, with a 1,5 cm to 3,5 cm thick layer of plaster at the bottom (Calcium sulphate (≥ 90%), manufacturer: Toupret, Plaster of Paris) (Fig 3b) moistened with facility water to achieve high humidity [34, 47, 48]. Animals were kept in incubators set to 10°C, 12°C or 15°C or at room temperature (20–22°C), to match the temperature of the natural habitat as much as possible. Photic conditions in which the animals were kept is given in S1 Table and S2 Appendix.

### Water preparation

Facility fresh water was prepared weekly in a 100 L plastic barrel, by mixing tap and reverse osmosis (RO) water (Amtra Osmosis System 190), to achieve electrical conductivity of 350–450 μS. Before use, the water was left for 48 hours for chlorine to evaporate. Water in the barrel was agitated and ran through a UV-C water sterilizer (JBL Procristal UV-C Compact plus 18 W) for 12 hours a day in 30-minute intervals. The barrel was thoroughly cleaned every 6 months with running hot water, 70% ethanol and alcoholic vinegar. Facility seawater (35‰) was prepared by mixing RO water and Sea Salt (Instant Ocean). The water was kept in 5 L containers and cooled in incubators to the required temperature before being used for water changes.

### Food

**Fig 4:**
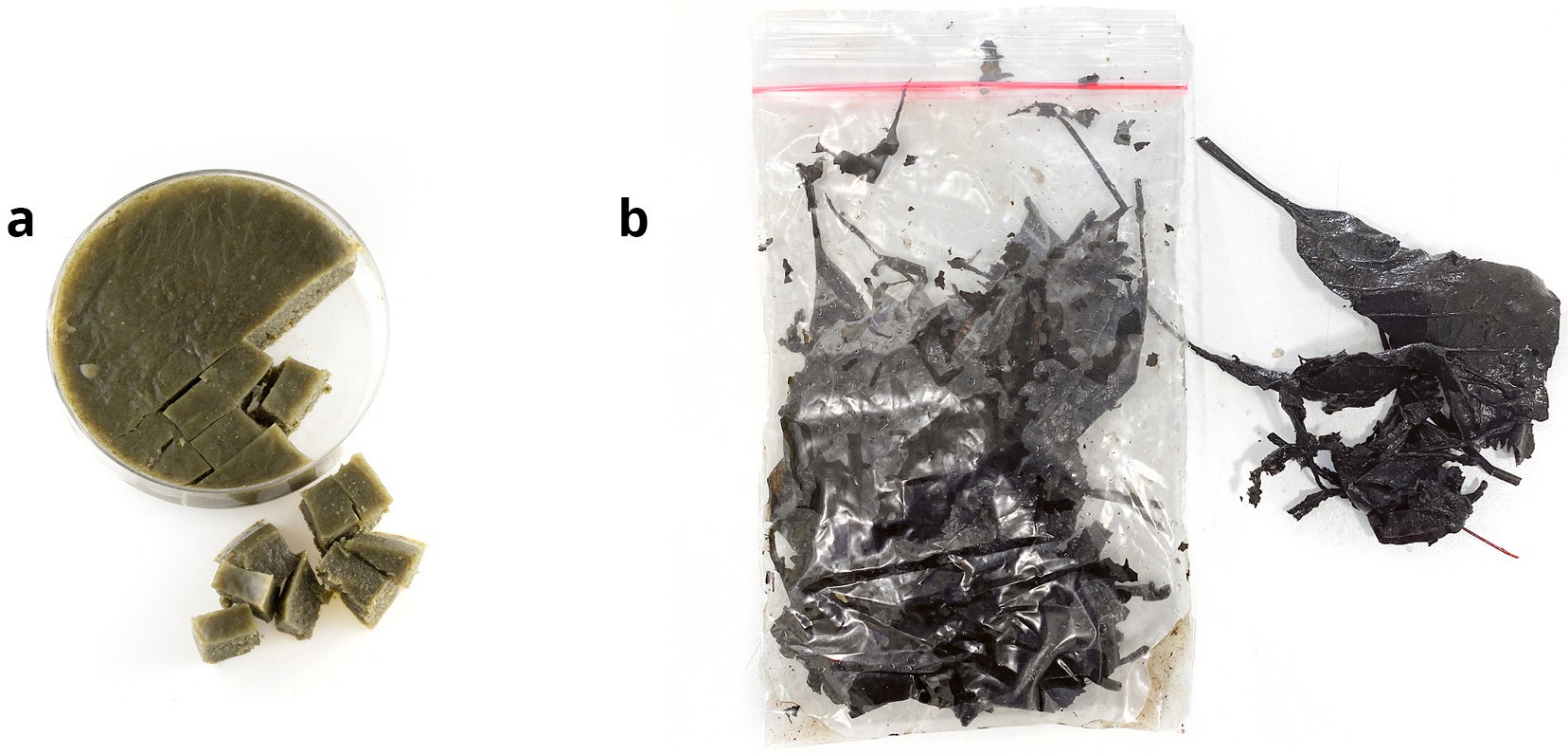
Food used in our studies. A. Food pellets cut into cubes. B. Conditioned leaves packaged in small bags are kept frozen until use. Credit: Tin Rožman.

Animals were fed with conditioned leaves [49] and/or food pellets (Fig 4). Fallen dry leaves of *Acer* sp. and *Alnus glutinosa* were collected in autumn, boiled to remove tannin (*Acer* sp.), autoclaved, and then conditioned in spring water for one to three weeks with aeration until they were covered with biofilm and slimy on touch. Conditioned leaves were kept in small batches at -20°C and thawed prior to feeding. Food pellets [47] consisted of a slurry of 5 g of finely grounded shrimp food (JBL NovoPrawn) in 100 ml of distilled water used to prepare a 3% agarose solution (Agarose SERVA Cat. No 11400, SERVA Electrophoresis GmbH). A 3–5 mm thick layer of the solution was poured into a Petri dish, left to solidify, and stored at -20°C. Prior to feeding, food pellets were thawed and cut into 3–5 mm squares. The amount of food was adjusted according to the species and number of individuals in a colony. Food pellets remained intact and were left for days in the containers without significant water fouling, ensuring the constant availability of food for the animals. Starvation procedure prior to specific experiments is described in S3 Appendix.

### Animal handling

Watercolor brushes, transfer pipettes, turkey basters, and soft tweezers were used for animal manipulation, depending on the species. Tools were sterilized with 70% ethanol and hot water between handling animals from different containers. When working with terrestrial isopods the air conditioning in the room was turned off to prevent desiccation.

### Cleaning and disinfection

Air recirculating UV-C devices (UVR-Mi, Biosan) were used for air sterilization (daily, during 8 working hours). Ceiling UV-C lamps (Osram AirZing PRO 5030) were used for general facility sterilization and turned on once a week for 30 mins, or more often in case of disease outbreaks. Disposable sticky floor mats were placed by the door for removing dirt and debris from shoes. Benches were cleaned with 70% ethanol after every use. Containers were cleaned in a 75°C dishwasher cycle with alcoholic vinegar added to prevent limescale buildup and two rinse cycles. The animal facility was kept free from all other disinfecting chemicals and detergents.

### Diseases and outbreaks

**Fig 5:**
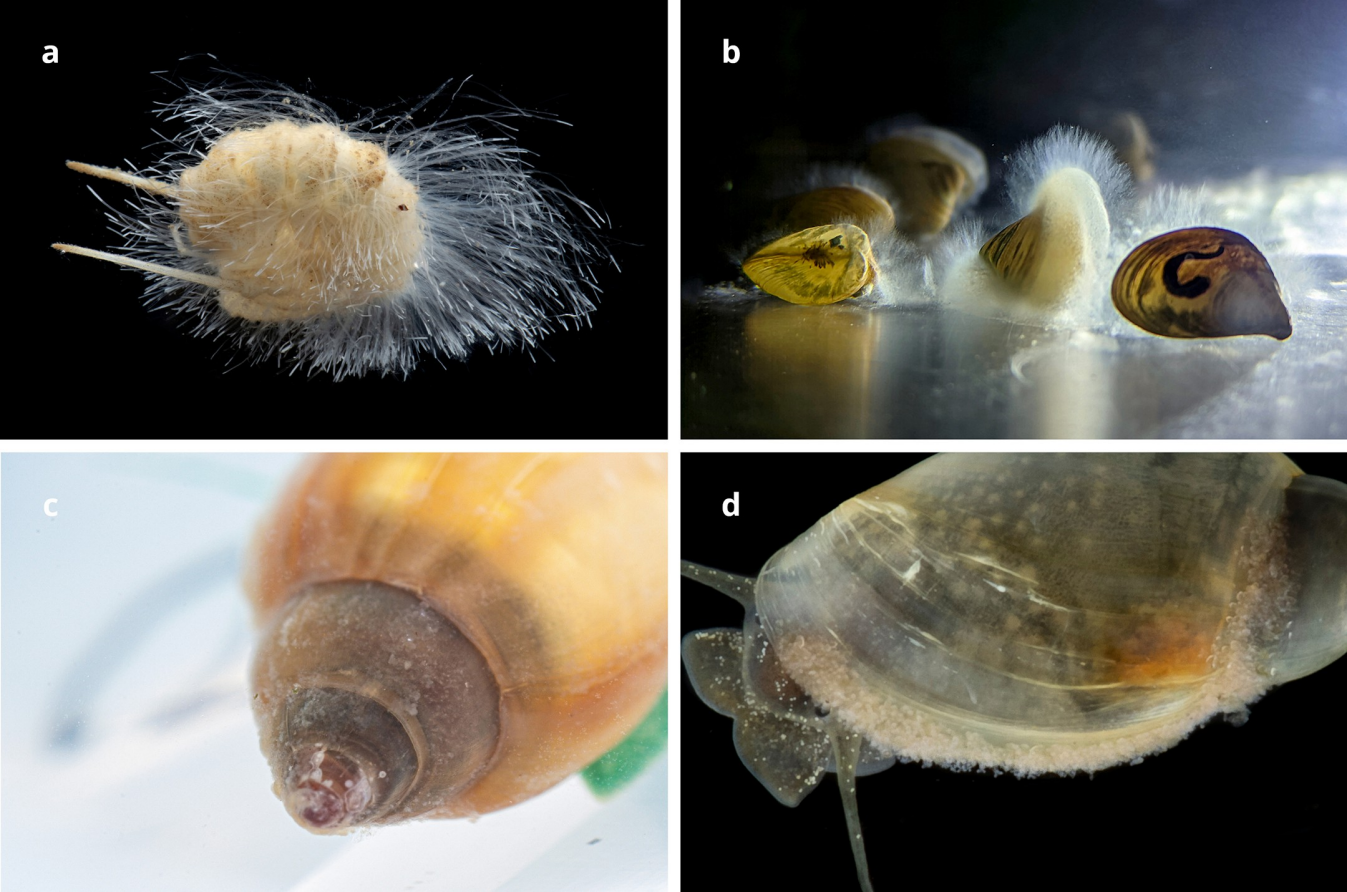
Diseases and affected colonies. A. Aquatic isopod *Monolistra radjai* with fungal overgrowth. B. Dreissenid bivalve (*Congeria* sp.) with fungal infestation. C. Physid snail (*Physella* sp.) with bacterial overgrowth on the shell. D. Physid snail (*Physella* sp.) with colonies of rotifers on the shell. Credit: Tin Rožman.

In case of microbial contamination, the affected population was immediately isolated from the rest of the colonies and quarantined outside the main facility to prevent cross-contamination. When this was not possible, a separate incubator in the facility or a lower shelf in an incubator was used to prevent transmission to other colonies. The incubators, containers, surfaces, and tools were sterilized with running hot water, 70% ethanol, UV-C lamps, and/or gamma radiation and the replaceable tools were discarded. All colonies of the infected species were inspected for the same problems. Depending on the type of infection, which may include fungal overgrowth (Fig 5a and 5b), bacterial outgrowth (Fig 5c), and the presence of other organisms such as protozoa or epibiontic/parasitic metazoans (Fig 5d), different treatments were applied. The first measures included increasing the frequency of water and container changes and sometimes a reduction in the amount of food. Several commercial antibiotics, fungicides, and drugs used in aquaculture of fish, crustaceans and mollusks were tested but without any success (e.g. SERA Bactopur, Ecocid S, eSHa 2000, Gentamycin, Rifampicine, Erithromycin, Vancomycin, Ciprofloxacin, Colistin, Acriflavine).

## Results

Over three years, we screened terrestrial and aquatic invertebrate species of different taxonomic groups from cave and surface habitats. Test colonies were brought to the laboratory and exposed to different culturing strategies including different types of food, housing, and temperature to produce breeding colonies. A complete list of species and sampling sites with comments on survival and culturing success per site is given in S1 Table. We established culturing protocols for aquatic sphaeromatid and asellid isopods and physid snails, as well as terrestrial trichoniscid isopods (Table 1). Below we present detailed guidelines for each of the groups.

### Trichoniscidae

*Housing*, *food and care*. We kept trichoniscids in containers with layer of plaster, at 12°C or 15°C, in densities of around 20 specimens per container. Smaller trichoniscids, *Alpioniscus balthasari* (Fig 2a) (body size 6 mm) were housed in round (d = 7cm) 200 mL plastic containers with a small water pool in the plaster. Containers were kept tightly closed to retain high humidity because all cave and many surface isopods are sensitive to low humidity. Larger, semiaquatic species *Titanethes albus* (Fig 2b) (body size 17mm) were housed in 1 L plastic containers with plaster inclined so half of the container was filled with water (Fig 3c). Because of these larger water pools, the containers did not need to be tightly closed to prevent desiccation of animals. A few sterilized small rocks from the collection site were embedded in the plaster layer to mimic the original habitat in some containers (Fig 3c). During regular maintenance, every two to four weeks, we moistened the plaster and removed dead animals. Trichoniscids were fed *ad libitum* with conditioned leaves. For handling, we used a fine wet brush for small specimens, and soft tweezers for larger ones.

*Culturing*. We maintained cave trichoniscids in colonies for several years. Juveniles of the two species were occasionally observed but did not survive to adulthood. We failed to culture surface trichoniscids (see S1 Table).

*Problems*. Containers occasionally became moldy, and the affected populations would start to decline. This problem occurred frequently in the initial setups for *Titanethes* (sometimes 50% of colonies) but only rarely for *Alpionisus*. Even after animals were transferred to new, clean containers and provided with fresh food, mold would return within a few weeks. This problem ceased after we provided housing containers with inclined plaster and large water pools that were not tightly closed.

### Asellidae

*Housing*, *food and care*. We housed asellids (Fig 2c–2e) in plastic containers of different sizes (1–3 L), in incubators at 12°C or 15°C, and densities of around 20–50 adults per container depending on the container size. *Caecidotea* were kept at 10–12°C. Conditioned leaves were always provided as food and shelter. We supplemented the diet of surface asellids with approximately 1 food pellet per 20 individuals once per month. They were also fed more food during the breeding period. Cave asellids ate less and were not given food pellets to avoid fouling of water. Juveniles fed on adult feces [50] along with conditioned leaves. During regular maintenance once a month, we changed about one-third of the water, provided new conditioned leaves, removed dead animals, as well as some bottom debris and excrement. For handling we used a brush, transfer pipette, or turkey baster, depending on the type of work and species size.

*Culturing*. We successfully maintained colonies of almost all asellids we attempted. Adults brought from the field usually survived at least a year. We established reproductive colonies for most *Asellus aquaticus* populations, as well as *Proasellus coxalis* s.l., *P*. *anophtalmus*, *P*. *hercegovinensis*, *Caecidotea kenki* and *C*. *pricei*. However, the rate of reproduction and juvenile growth in cave *Proasellus* species was not sufficient for establishment of a self-sustaining colony. Reproduction in cultures of *Asellus aquaticus* and *Proasellus coxalis* s.l. peaked from February till May. We separated generations by moving ovigerous females or pairs in amplexus to dedicated containers not smaller than 40 ml and isolating the juveniles afterward. We tried separating juveniles using breeding containers with a nylon mesh bottom (0,5x0,5 mm) [50] (S3 Appendix), but they did not separate generations completely as not all juveniles fell through the mesh to the rearing container. Survival of some asellids depended on the sampling locality. *Asellus aquaticus* from Planina passage in the Postojna-Planina cave system (Slovenia) survived for only two months.

*Problems*. The water in the containers would get cloudy or fouled in about 10% of colonies at any given time. This was not lethal to the animals in the short term and doubling the frequency of water changes solved the problem. Other problems we encountered were the appearance of a biofilm on the water surface, slime in the water, and mold on the lids. We removed any surface biofilm with a paper towel and then changed half of the water. If the water became slimy, we transfered the colony to a new container with fresh facility water and food. Mold on the container edges and the lids was wiped off, and if it persisted, animals were transferred to a new clean container. Some colonies were contaminated with epibiotic rotifers, but their survival was unaffected.

### Sphaeromatidae

*Housing*, *food and care*. We kept about 20 sphaeromatids per 1 L plastic containers in incubators. Cave species (*Monolistra pretneri* and *M*. *velkovrhi*) (Fig 2f) lived at 12°C in facility freshwater and surface marine species (*Lekanesphaera hookeri*) (Fig 2g) at 15°C in facility seawater. They were fed *ad libitum* with conditioned leaves and provided food pellets once a month, approximately 1 pellet per 20 individuals. About one third of the water was replaced every two weeks for the freshwater species, and water was completely changed every week for the marine species. For handling we used small transfer pipette or turkey baster.

*Culturing*. All sphaeromatids survived for more than one year in the laboratory. Although ovigerous females of *Lekanesphaera hookeri* were recorded in the laboratory, no juveniles survived to adulthood. We observed that starvation and stress triggers mating in *L*. *hookeri*. Cave *Monolistra* species did not reproduce.

*Problems*. We had problems with colonies of *Monolistra* on a couple of occasions because of an overgrowth of white filamentous infestation which interfered with locomotion (Fig 5a), and the colonies eventually died off despite increased water changes. But overall, colonies of sphaeromatids were cultured without major problems.

### Physidae

*Housing*, *food and care*. *Physella* snails (Fig 2h) lived at room temperature (20–22°C) in facility water at densities of up to 10 and 25, in 200 mL and 1 L plastic containers, respectively. Alternatively, setup in the Weck lab consisted of 3.8 L small plastic aquariums (Top Fin Diamond) with up to 50 individuals fitted with small internal fiber filters and water from a nearby cave. The smaller containers allow easier handling, and the larger aquariums require less hands-on time for maintenance, but both are good options. Additionally, in case of disease outbreak snails in smaller containers were easily screened and isolated. For isolating individuals, containers of at least 30 mL capacity were needed to avoid fouling of the water and adhesion of debris on the snail shells. Water was changed completely 2–3 times a week for small containers and every two weeks for 3.8 L aquaria. One food pellet per 10 individuals was given after each water change in containers or grounded Tetra Pleco Wafers in quantity of 1 wafer per 3.8 L aquarium per week.

*Culturing*. We were successful in setting up colonies from Fogelpole cave from which physids are easy to culture, proliferative, and with short generation time. Snails of approximately 55 days in age started deposited eggs on the walls of the container up to several times a week. Eggs were usually left to hatch and grow in the same container as adults. If examination of eggs was needed, separating them from the container wall with a thin blade and moving to another container resulted in poor hatching rates or the death of young snails. A better approach was to keep adult snails in thin plastic cups, then cutting out the part of the cup with deposited eggs and moving it to a new container. Juvenile snails were also fed with the food pellets or grounded Pleco Wafers.

*Problems*. Occasionally, some of the snails or colonies were affected by overgrowth on the shells (Fig 5c). The infection changed the behavior of the snails—they moved more slowly, spent more time withdrawn in the shell and avoided food. Infected specimens usually died, although some recovered. These problems were more common in smaller containers with insufficient water per snail ratio and if the snails were fed food that quickly dissolved, contaminating the water. We found that food pellets and Tetra Pleco Wafers were the least problematic and did not contaminate the water. The overgrowth contamination was mitigated with frequent removal of uneaten food and feces, along with quarantine of infected snails.

## Discussion

It is becoming increasingly clear that traditional model organisms are insufficient to answer a variety of questions of interest to modern biological sciences [51, 52]. An emerging group of non-traditional and atypical model organisms are cave animals [12]. They are promising candidates for finding answers to some of the long-standing questions in evolutionary biology but also possible solutions to a variety of problems that impact human health. Their phenotypes, such as albinism, eye degeneration, fat deposition, and immune system changes have converged independently across different animal phyla, offering comparative research models that enable a deeper understanding of the underlying genetic, physiological, and evolutionary mechanisms driving these adaptations in disparate lineages. Interestingly, the same traits also mirror symptoms of many human diseases, yet these species thrive without obvious health defects [18, 20]. Because caves are often technically challenging to access, and population densities of cave species are generally low [33], comprehensive studies of these species require an *ex situ* approach.

Laboratory conditions provide a high degree of control over experiments, and the use of species with established husbandry and breeding protocols greatly increases reproducibility as well as the number of tools, methods, and experiments that can be used for scientific studies [53]. However, there are no standardized husbandry and breeding protocols for cave animals (except for the Mexican cavefish, discussed below), and even the most basic guidelines on how to keep the cave animals in the laboratory are scarce [54]. Maintaining cave organisms in laboratory cultures is not trivial, because the same special characteristics that make them interesting model systems for research also bring disadvantages to rearing them in the laboratory. For example, the known direction of evolutionary change from the ancestral surface to a derived cave species enables a direct comparison to reveal genetic, ecological and evolutionary processes that led to the appearance of certain phenotypes [55]. To study this, both cave and surface relatives must thrive under laboratory conditions. However, cave animals are highly specialized and often less resistant to environmental fluctuations due to the buffered climatic conditions of the subterranean environment [34, 56]. Therefore, surface and cave species pairs, despite being closely related, may require different conditions in terms of temperature, photoperiod, humidity, food, and might have different susceptibilities to diseases which complicates their maintenance in the laboratory. However, not all cave species are necessarily difficult to culture. For example, the Mexican cavefish *Astyanax mexicanus*, the best-known cave model species, thrive and regularly spawn under conditions that do not resemble the cave environment (normal light cycle, abundant food, etc.) [5]. Because of the successful breeding protocol, the scientific community interested in Mexican cavefish is growing rapidly [57]. A search for "cavefish" or "cave fish" on the Web of Science yields 1691 articles, 475 of which deal with *Astyanax* (evaluated on February 6, 2024), and the 10 most productive years are all after 2013. The rise of the *Astyanax* system benefited greatly from the direct applicability of protocols and methods developed for the relatively closely related zebrafish model [58]. Clearly, successful maintenance and breeding protocols are a critical milestone that establish a particular species as a model organism, promotes scientific interest and increases the use of that species in research.

The list of cave invertebrates used as models is limited [12] and is dominated by crustaceans. The isopod crustacean *Asellus aquaticus* is the most commonly used because of the ability to complete its life cycle under laboratory conditions [59, 60]. Studies of *Asellus* cave populations yielded several important insights into its evolution, behavior, as well as the molecular basis for some of its phenotypes [60, 61]. Also, its surface morphotype is bred for laboratory experiments, such as ecotoxicology [62], for biological control in aquaculture [63], but also as aquarium pets, or food for aquarium fish. Subterranean crustaceans from several groups, mostly copepods, amphipods and isopods, have been used for ecotoxicity studies (for review see 54), but the tests were conducted on field-collected animals and only within a few days of collection, so no culturing was necessary. Attempts to use some of the other species (e.g. isopod genus *Proasellus*) for experimental studies have failed so far due to difficulties in culturing (pers. info. Florian Malard). Another group of organisms, physid snails, have been extensively used in laboratory experiments [64–66], although only a few studies have been published on species from caves [67, 68].

Following the successful example of the *Astyanax* system and the promising *Asellus* model, we identified various cave and surface species from different taxonomic groups as candidates for laboratory cultures. Aquatic physid gastropods (*Physella*), sphaeromatid (*Lekanesphaera* and *Monolistra*) and asellid isopods (*Asellus*, *Proasellus*, and *Caecidotea)*, and the terrestrial trichoniscid isopods (*Alpioniscus and Titanethes*) thrived under laboratory conditions, with some asellids and physids reproducing regularly and giving rise to several generations in the laboratory (Table 2). Regular maintenance and inspection was shown to be a prerequisite for healthy and long-lived laboratory colonies. Running a facility with diverse animal groups is labor and time consuming, therefore we tried to optimize hands-on time (Table 1) and unify culturing conditions for different species to a certain extent. For example, our facility water is suitable for aquatic species of different taxonomic groups and different cave and surface habitats. We decided against using water collected from natural springs, and instead invested the time into producing custom facility water to prevent introducing other species or water contaminants into our facility. Further, we have optimized the production of food pellets, with physical consistency that prevents their disintegration and fouling the water over lengthy periods. The composition of our food pellets ensures constant availability of high nutrient food for species from diverse groups. All this simplifies and reduces staff labor and increases the degree of control of laboratory stock of animals and consequently over experiments and their reproducibility.

Initially, we have identified and tried to culture more cave-surface species pairs from other taxonomic groups. These include cave (*Congeria*) and surface (*Dreissena*) dreissenid bivalves, planorbid gastropod (*Ancylus*), cave (*Monolistra radjai* Prevorčnik & Sket, 2007) and surface (*Trichoniscus matulici* Verhoeff, 1901 and *Proasellus karamani* from various sites) isopods, and cave (*Troglocaris*) and surface (*Atyaephyra*) freshwater atyid shrimp (S1 Table). They could not be successfully cultured, although not the same efforts were made for all of them. We gave up on some of the species early on (*Ancylus*, atyid shrimps) as they had more frequent disease outbreaks, or low survival. On the other hand, we devoted intense several-year-long efforts into establishing cultures and optimizing lab conditions for dreissenid bivalves. More than a dozen different water parameters were tested, the type of food and frequency of feeding, different housing options, with and without different types of substrates, water aeration and filtration. However, the cave bivalves *Congeria kusceri* Bole, 1962 and *Congeria jalzici* Morton & Bilandžija, 2023 were repeatedly overgrown by a fungus (a new species for science, pers. comm. I. Kušan and N. Matočec), despite all our efforts, including treatments with antimycotics and antibiotics, or introducing other species to the tanks (e.g. physid snails, shrimps) to control the fungus growth. The surface species *Dreissena polymorpha* (Pallas, 1771) had a better survival rate, but never reproduced and the colonies eventually collapsed. We also kept the terrestrial trichoniscid surface species *Trichoniscus matulici* for a year, but their colonies were constantly infected by mold and collapsed despite our efforts. Another *Trichoniscus* species, *T*. *pussilus*, is known to reproduce in laboratory cultures [69], but we have not found a solution for culturing *T*. *matulici*. Perhaps our target species, living in very moist moss on stones in and along the riverbank, is too specialized for cultivation.

Cultivation success in the laboratory did not seem to be only species specific but also depended on the collecting-site. Populations of some species sampled from one site thrived, but from another site would collapse in a couple of months (see S1 Table). For example, physid snails from Fogelpole Cave (USA) thrive in laboratory cultures, but a population from the nearby Illinois Caverns, as well as some related species collected in Croatia performed very poorly. Also, cave *Asellus aquaticus* were more difficult to maintain than surface morphotypes, consistent with previous studies [70]. It is unknown why some of our species or populations performed poorly in laboratory cultures, but it is possibly due to sampling season or presence and susceptibility to diseases. In general, establishing a successful maintenance and breeding protocol is preceded by a lengthy trial and error period. The list of possible improvements is extensive and greatly depends on the available staff time and resources. Possible directions which might bring further improvements and increase cultivation success include testing of different cultivation substrates for terrestrial species, introduction of additional species into culturing containers (e.g. Daphnia¸ Collembola) that would feed on, or compete for food resources with undesirable organisms (protozoa or fungi), building a flow-through housing system for the troublesome cave aquatic invertebrates, etc.

In addition to the usual issues to consider when establishing laboratory cultures of wild animals, such as housing, feeding, and care, there are additional challenges with cave animals. Adaptations to caves often include slow metabolism and growth as well as low reproductive rate, the opposite of what is required of a laboratory model organism [71]. This is also reflected in our study, as most of the cave-adapted species in our facility did not reproduce or reproduced at lower rates than required for a self-sustaining colony. However, we have been successful in maintaining them in our laboratory cultures for extended periods, allowing for their repeated use for various experiments which reduced their sampling in the wild. Moreover, successful maintenance in laboratory cultures is the first and critical step in the process of adaptation of wild animals to captive breeding. Because of the similarity of the major ecological characteristics of subterranean ecosystems where different species use similar energy resources, and develop similar adaptations, the guidelines and recommendations presented here could be applied to a variety of other species of aquatic and terrestrial invertebrates. Our experience and recommendations therefore represent an important milestone for further development of protocols for these and other species in the expanding field of cave biology, and for the use of cave animals as model organisms in biomedicine, functional ecology, evo-devo and neuroethology, among other disciplines.

## Supporting information

S1 Appendix Details on the distribution, habitat, ecology and morphology of cave and surface invertebrates that were successfully maintained in the laboratory for this study. (DOCX)

S2 Appendix Photic conditions of the facility. (DOCX)

S3 Appendix Emptying the gut content before experiments. (DOCX)

S1 Table List of species used in this study, sampling sites, collection methodology, their habitat, rearing conditions and survival and culturing success in the laboratory. Scores low (L), medium (M), and high (H) for survival of the wild individuals in the lab and for survival of offspring denotes <20%, 20–80% and >80% of individuals surviving, respectively. Frequency of reproduction is defined as >1 or <1 per year. Photic conditions are abbreviated as LD (12:12 h light-dark photoperiod), DD (constant darkness), WL (incubators without lighting system) and EE (photoperiod of the external environment) (see S2 Appendix for description of photic conditions). Where no reproduction is reported, this means that no mating or embryos were observed.
(DOCX)
