# Supplementary material for: A practical guide for the husbandry of cave and surface invertebrates as the first step in establishing new model organisms
Source: PLoS One. 2024 Apr 4;19(4):e0300962. doi: 10.1371/journal.pone.0300962 (PMC10994295; doi:10.1371/journal.pone.0300962)
Supplement: S1 Appendix — (DOCX) [file pone.0300962.s001.docx]

**A practical guide for the husbandry of cave and surface invertebrates as the first step in establishing new model organisms**

Marko Lukić, Lada Jovović, Jana Bedek, Magdalena Grgić, Nikolina Kuharić, Tin Rožman, Iva Čupić, Bob Weck, Daniel Fong, Helena Bilandžija

S1 Appendix

**Details on the distribution, habitat, ecology and morphology of cave and surface invertebrates that were successfully maintained in the laboratory for this study.**

Asellidae

Asellidae is a family of freshwater isopod crustaceans. *Proasellus coxalis* s.l. is a surface species, which probably originates from the Middle East, northern Africa, and southern Europe and has spread northwards to Scandinavia during the 20^th^ century (1). It is morphologically and genetically very diverse, and probably includes several different species (2,3), therefore we use the name in the broad sense. As a part of the benthos community it inhabits different freshwater habitats, as well as subterranean ones (3) and can tolerate brackish water (1). The body is grayish/brownish to pale (even colorless) with well-developed eyes, females are up to 8 mm and males up to 10 mm in length (4). *Proasellus karamani,* a surface species distributed in Montenegro, Bosnia and Herzegovina, Serbia and Macedonia (5) inhabits springs and streams. The body colour is brown and it has normally developed eyes. *Proasellus anophtalmus* is a cave species, widely distributed in the southeastern part of Dinaric Karst. It inhabits different subterranean habitats, including small cracks and epikarst, while in caves it can be found in small drip ponds (6). It is eyeless and depigmented, with narrow body up to 3,9 mm length in females and 4,5 mm in males (7). The other cave *Proasellus* species, *P. hercegovinensis* is also depigmented and without eyes, but with larger body where males are up to 10 mm in length. It has a narrower distribution in Popovo polje in Herzegovina but probably inhabits larger subterranean water bodies (6). *Asellus aquaticus* is a widely distributed surface species in Europe, with several cave and cave-like populations. It is relatively large isopod, where females are up to 15 mm and males up to 20 mm in length (4). Surface populations are usually brown, but depending on the substrate and vegetation can exhibit pale body colors. Cave populations are eyeless and depigmented, while cave-like populations show extensive individual variation in body pigmentation and eyes development (8). *Caecidotea kenki* is a surface species common in seepage springs, exit points of shallow groundwater from hypotelminorheic habitats (9,10), along the Potomac River near Washington, D.C., USA. It has a grayish body about 6-8 mm long and 2 mm wide, and well-developed eyes. *Caecidotea pricei* is an eyeless and depigmented cave species, up to 10 mm long and 2-3 mm wide, with an extensive range in the Shenandoah Valley of the eastern USA (11).

Sphaeromatidae

Sphaeromatidae is a family of isopod crustaceans which live in marine, brackish and freshwater habitats. *Lekanesphaera hookeri* geographic range extends from SE Sweden throughout Europe to the whole Mediterranean Sea. It is a marine benthic isopod that also lives in brackish habitats and generally is found close to the shore (12). It is grayish with well-developed eyes. The body size is up to 7 mm in females and 11 mm in males (12). The genus *Monolistra* is distributed in Dinaric Karst and Alps. All species are exclusively subterranean, eyeless and depigmented, usually living in cave lakes and running waters (13). They differ in cuticle texture and body size. *Monolistra velkovrhi* (smooth body, up to 6,5 mm in length) is distributed in the continental part of Slovenia and Croatia (13). Both, *M. pretneri* (smooth to the nodulated body, up to 10 mm in length) and *M. radjai* (smooth body with well-developed uropodes, up to 8,6 mm in length) are endemic for Croatia and distributed near the Adriatic coast (14,15). *Monolistra radjai* inhabits anchialine caves and is the only known *Monolistra* species which tolerates brackish water (15).

Trichoniscidae

Trichoniscidae is a family of terrestrial isopods. *Trichoniscus matulici* is a widespread surface species, distributed in southern Europe (16). It is highly hydrophilic and can be found near water bodies (17). It is up to 4 mm long, grayish/brownish with well developed eyes. In Dinaric Karst cave populations exist and are less pigmented and show signs of eye degeneration (18). Both terrestrial cave species, *Titanethes albus* and *Alpioniscus balthasari* are eyeless, depigmented and semiaquatic, and can spend some time in the water during unfavorable conditions (19,20). *Titanethes* *albus* is large cave species, up to 17 mm in length, and distributed in the northwestern part of Dinaric Karst, from Italy to Bosnia (21), while *A.* *balthasari* is smaller species, up to 6 mm in length, and distributed in Dalmatia and Herzegovina (19).

Physidae

Physidae is a worldwide distributed family of freshwater, pulmonated snails, within Lymnaeoidea superfamily. They originated from North America. *Physa acuta* is an invasive species which has spread on all continents (22). Physid snails have sinistral shells of up to 2 cm in size and lack the operculum. They are outcrossing simultaneous hermaphrodite (23) with a high reproduction rate.

Populations of cave-dwelling physids are found in the US. They include several populations in caves in Illinois - Fogelpole Cave, Stemler Cave, and Illinois Caverns, populations in several Ozark caves in Missouri, and a population of endemic *Physa spelunca* in sulfidic Lower Kane Cave, Wyoming (24).

References:

1. Kemp JL, Ballot A, Nilssen JP, Spikkeland I, Eriksen TE. Distribution, identification and range expansion of the common Asellidae in Northern Europe, featuring the first record of Proasellus meridianus in the Nordic countries. Fauna Norv. 2020 Aug 12;40:93–108.

2. Ketmaier, Argano, Cobolli, De Matthaeis, Messana. A systematic and biogeographical study of epi- and hypogean populations of the Proasellus species group from Sardinia, central Italy and Jordan: allozyme insights. J Zoological System. 2001 Mar 22;39(1–2):53–61.

3. Stoch F, Valentino F, Volpi E. Taxonomic and biogeographic analysis of the Proasellus coxalis-group (crustacea, isopoda, asellidae) in Sicily, with description of Proasellus montalentii n. sp. Hydrobiologia. 1996 Jan;317(3):247–58.

4. Wouters K, Vercauteren T. Proasellus coxalis sensu auct. (Crustacea, Isopoda) in a lowland brook in Heist-op-den-Berg: first record in Belgium. Lauterbornia. 2009;67:53–61.

5. Sket B. Crustacea Isopoda (aquatica). Catalogus faunae Jugoslaviae, Cons Acad Sc RPSF Jugoslaviae. 1967;3:1–21.

6. Henry JP, Lewis JJ, Magniez GJ. Isopoda: Asellota, Aselloidea, Gnathostenetrioidoidea, Stenetrioidoidea. In: Botosaneanu L, editor. Stygofauna Mundi. Leiden: E.J. Brill; 1986. p. 434–64.

7. Karaman SL. Asellus cavaticus Schiödte und seine Nächstverwandten. Acta Musei Macedonici Scientiarum Naturalium. 1955;3(12):1–40.

8. Protas M, Trontelj P, Prevorcnik S, Fišer Ž. The Asellus aquaticus species complex: an invertebrate model in subterranean evolution. In: Malard F, Griebler C, Rétaux S, editors. Groundwater ecology and evolution [Internet]. Second edition. S.l.: Academic Press; 2023. p. 329–50.

9. Culver DC, Holsinger JR, Feller DJ. The Fauna of Seepage Springs and Other Shallow Subterranean Habitats in the Mid-Atlantic Piedmont and Coastal Plain. Northeastern Naturalist. 2012 Aug;19(mo9):1–42.

10. Culver DC, Pipan T. Shallow Subterranean Habitats: Ecology, Evolution, and Conservation [Internet]. Oxford University Press; 2014 [cited 2023 Sep 21]. Available from: https://academic.oup.com/book/26534

11. Holsinger JR, Culver DC. The Invertebrate Cave Fauna of Virginia and a Part of Eastern Tennessee: Zoogeography and Ecology. Brimleyana. 1988;14:1–162.

12. Potenza L, Mancinelli G. Body mass-related shift in movement behaviour in the isopod *Lekanesphaera hookeri* (Isopoda, Flabellifera): A laboratory study. Italian Journal of Zoology. 2010 Aug 26;77(3):354–61.

13. Sket B. Isopoda: Sphaeromatidae. In: Botosaneanu L, editor. Stygofauna Mundi. Leiden: E.J. Brill; 1986. p. 423–7.

14. Prevorčnik S, Verovnik R, Zagmajster M, Sket B. Biogeography and phylogenetic relations within the Dinaric subgenus *Monolistra* (*Microlistra*) (Crustacea: Isopoda: Sphaeromatidae), with a description of two new species. Zoological Journal of the Linnean Society. 2010 May;159(1):1–21.

15. Prevorčnik S, Sket B. An ecologically peculiar new species of Monolistra (Crustacea: Isopoda: Sphaeromatidae) from cave waters in the Dinaric karst of Croatia. Subterranean Biology. 2007;5(60):23–7.

16. Schmalfuss H. World catalog of terrestrial isopods (Isopoda: Oniscidea). Stuttgarter Beiträge zur Naturkunde. 2003;654:1–341.

17. Verhoeff KW. Über paläarktische Isopoden (4. Aufsatz). Zoologischer Anzeiger. 1901;24(66–79).

18. Buturović A. O nekim vrstama roda Trichoniscus iz Jugoslavije. Godišnjak biološkog instituta u Sarajevu. 1955;8:105–11.

19. Bedek J, Taiti S, Bilandžija H, Ristori E, Baratti M. Molecular phylogeny and taxonomy of troglobiotic *Alpioniscus* (*Illyrionethes*) species from the Dinaric Karst (Isopoda: Trichoniscidae). Zoological Journal of the Linnean Society. 2019;20:1–46.

20. Strouhal H. *Titanethes* Schiödte. (Landasseln aus Balkanhöhlen in der Kollektion “Biospeologica balcanica” von Prof. Dr. Absolon. 6. Mitteilung.). Studien aus dem Gebiete der allgemeinen Karstforschung, der wissenschaftlichen Höhlenkunde, der Eiszeitforschung und den Nachbargebietzen, Biologische Serie. 1939;5:1–34.

21. Karaman IM, Horvatović M. Revision of the genera *Cyphonethes* Verhoeff, 1926 and *Titanethes* Schioedte, 1849 (Isopoda: Oniscoidea: Trichoniscidae) with a description of a new genus and three new taxa. Zootaxa. 2018 Aug 15;4459(2):261–84.

22. Van Leeuwen CHA, Huig N, Van Der Velde G, Van Alen TA, Wagemaker CAM, Sherman CDH, et al. How did this snail get here? Several dispersal vectors inferred for an aquatic invasive species: Dispersal of aquatic snails by several vectors. Freshwater Biology. 2013 Jan;58(1):88–99.

23. Wethington A, Dillon RTJ. Reproductive development in the hermaphroditic freshwater snail *Physa* monitored with complementing albino lines. Proc R Soc Lond B. 1993 May 22;252(1334):109–14.

24. Turner RD, Clench WJ. A new blind *Physa* from Wyoming with notes on its adaptation to the cave environment. Nautilus. 1974;88:80–5.
