# Supplementary material for: A practical guide for the husbandry of cave and surface invertebrates as the first step in establishing new model organisms
Source: PLoS One. 2024 Apr 4;19(4):e0300962. doi: 10.1371/journal.pone.0300962 (PMC10994295; doi:10.1371/journal.pone.0300962)
Supplement: S2 Appendix — (DOCX) [file pone.0300962.s002.docx]

**A practical guide for the husbandry of cave and surface invertebrates as the first step in establishing new model organisms**

Marko Lukić, Lada Jovović, Jana Bedek, Magdalena Grgić, Nikolina Kuharić, Tin Rožman, Iva Čupić, Bob Weck, Daniel Fong, Helena Bilandžija

S2 Appendix

**Photic conditions of the facility**

Our invertebrate facility is organized by photic conditions. There is a dark room where animals are kept in constant darkness in a separate room. To ensure complete darkness, doors were on sliding tracks and fitted into the grooves in the walls, with an additional curtain made of blackout fabric that covered the entire entrance door and wall. All the screens and LEDs on the electronic devices used in the dark room were turned off or taped. In the dark room only headlamps with dim red light were used while working with animals. The other part of the facility had natural diurnal light/dark photoperiod which followed the day and night outside. However, for the experiments where we control the light/dark regime we designed and mounted a custom-made lighting system with high quality LED lights (Gen 7 V10 Thrive Array, Bridgelux) and a flicker free dimmer in the incubators. Special attention was given to the position of the LEDs in the incubator and their passive heat sink mounted on the incubator walls that prevent warming of the nearby animal containers. Animals that did not require controlled photic regime were kept in incubators without lighting systems and light is on only when the door is opened. Physid snails are exposed to photoperiod of the external environment and dim light from headlamps only during handling.
