# Supplementary material for: A practical guide for the husbandry of cave and surface invertebrates as the first step in establishing new model organisms
Source: PLoS One. 2024 Apr 4;19(4):e0300962. doi: 10.1371/journal.pone.0300962 (PMC10994295; doi:10.1371/journal.pone.0300962)
Supplement: S3 Appendix — (DOCX) [file pone.0300962.s003.docx]

**A practical guide for the husbandry of cave and surface invertebrates as the first step in establishing new model organisms**

Marko Lukić, Lada Jovović, Jana Bedek, Magdalena Grgić, Nikolina Kuharić, Tin Rožman, Iva Čupić, Bob Weck, Daniel Fong, Helena Bilandžija

S3 Appendix

**Emptying the gut content before experiments**

For specific experiments that require empty gut content to avoid contamination, animals were kept in starvation. Aquatic isopods were starved in containers with the bottom replaced by nylon mesh (0,5x0,5 mm), stacked into a second same size container (McCahon and Pascoe 1988) (Supplementary Figure 1). This setup prevented animals from feeding on their own feces, however emptying the gut was not completely successful and occasionally gut content was visible even after several weeks of starvation.


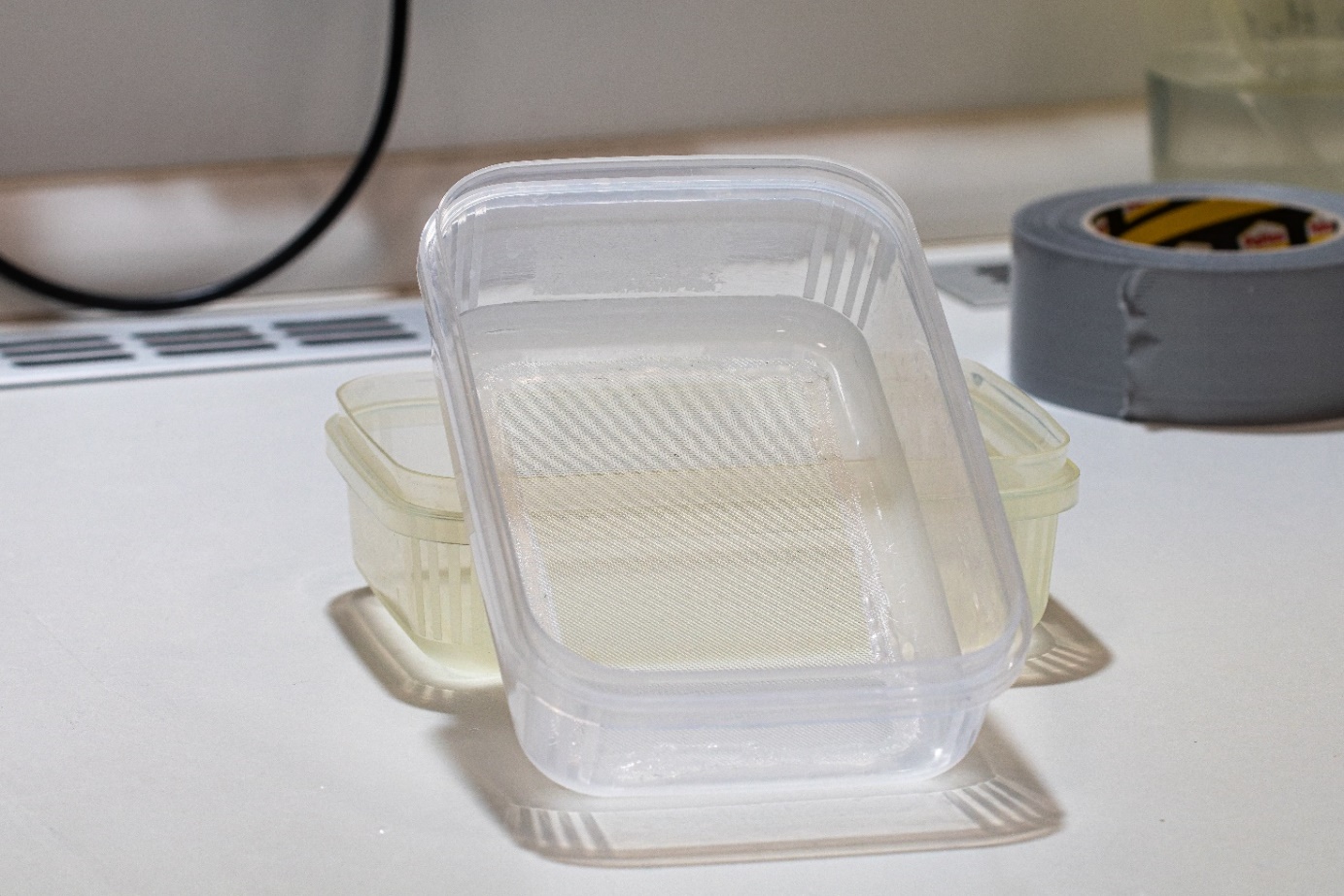


Figure 1. Setup used for isolating aquatic crustaceans from their feces with the purpose of emptying the gut for the experiments and for separating generations.
