# Supplementary material for: A practical guide for the husbandry of cave and surface invertebrates as the first step in establishing new model organisms
Source: PLoS One. 2024 Apr 4;19(4):e0300962. doi: 10.1371/journal.pone.0300962 (PMC10994295; doi:10.1371/journal.pone.0300962)
Supplement: S1 Table — Scores low (L), medium (M), and high (H) for survival of the wild individuals in the lab and for survival of offspring denotes <20%, 20–80% and >80% of individuals surviving, respectively. Frequency of reproduction is defined as >1 or <1 per year. Photic conditions are abbreviated as LD (12:12 h light-dark photoperiod), DD (constant darkness), WL (incubators without lighting system) and EE (photoperiod of the external environment) (see S2 Appendix for description of photic conditions). Where no reproduction is reported, this means that no mating or embryos were observed. (DOCX) [file pone.0300962.s004.docx]

**A practical guide for the husbandry of cave and surface invertebrates as the first step in establishing new model organisms**

Marko Lukić, Lada Jovović, Jana Bedek, Magdalena Grgić, Nikolina Kuharić, Tin Rožman, Iva Čupić, Bob Weck, Daniel Fong, Helena Bilandžija

**S1 Table.** **List of species used in this study, sampling sites, collection methodology, their habitat, rearing conditions and survival and culturing success in the laboratory.** Scores low (L), medium (M), and high (H) for survival of the wild individuals in the lab and for survival of offspring denotes <20%, 20-80% and >80% of individuals surviving, respectively. Frequency of reproduction is defined as >1 or <1 per year. Photic conditions are abbreviated as LD (12:12 h light-dark photoperiod), DD (constant darkness), WL (incubators without lighting system) and EE (photoperiod of the external environment) (see S2 Appendix for description of photic conditions). Where no reproduction is reported, this means that no mating or embryos were observed.

| **Group/ species** | **Sampling site** | **Collecting methodology** | **Species habitat** | **Water type / temperature in the lab (°C)** | **Photic condition in the lab** | **Survival of the wild individuals in the lab** | **Frequency of reproduction** | **Survival of offspring** | **Additional notes** |
| --- | --- | --- | --- | --- | --- | --- | --- | --- | --- |
| Trichoniscidae |  |  |  |  |  |  |  |  |  |
| *Titanethes albus* (C. Koch, 1841) | Tounjčica, Tounj (CRO) | Hand sampling (tweezers) | terrestrial / cave | NA / 12 | DD/WL | M | NA | NA | Survived for more than one year without problems. No reproduction. |
| *Alpioniscus balthasari* (Frankenberger, 1937) | Miljacka II, Oklaj, Knin (CRO) | Hand sampling (tweezers, aspirators) | terrestrial / cave | NA / 12 | DD/WL | H | NA | NA | Survived for more than one year without problems. No reproduction. |
| *Trichoniscus matulici* Verhoeff, 1901 | Ljuta, Konavle, Dubrovnik (CRO) | Hand sampling (brushes, tweezers, aspirators) | terrestrial / rocks near the surface stream | NA / 15 | LD/DD/WL | M | NA | NA | Survived for 6-9 months, after which the populations started to decline and eventually died off. We had problems with mouldy containers. No reproduction |
| *Trichoniscus matulici* Verhoeff, 1901 | Vilina špilja-Izvor Omble, Dubrovnik (CRO) | Hand sampling (brushes, tweezers, aspirators) | terrestrial / cave | NA / 15 | LD/DD/WL | M | NA | NA | Survived for 3-6 months, after which the populations started to decline and eventually died off. We had problems with mouldy containers. No reproduction |
| Asellidae |  |  |  |  |  |  |  |  |  |
| *Caecidotea kenki* (Bowman, 1967) | Pimmit Run seepage spring, Arlington, VA (USA) | Hand sampling (turkey baster) | aquatic / seepage spring | freshwater / 10 - 12 | DD | H | >1 | M | Colonies maintained for over 3 years, with total mortality of 35%. Juveniles reach adulthood and multiple lab generations have been raised thus far. |
| *Caecidotea pricei* (Levi, 1949) | Ogden Cave, Shenandoah County, Virginia (USA) | Hand sampling (turkey baster) | aquatic / cave | freshwater / 10 - 12 | DD | M | <1 | L | Colonies maintained for over 2 years, with total mortality of 60%. Juveniles reach adulthood and one lab generations have been raised thus far. |
| *Asellus aquaticus* (Linnaeus, 1758) | Sušik, Drežnica, Brinje (CRO) | Hand sampling (turkey baster); aquatic D-net | aquatic / surface stream | freshwater / 12 | LD/DD | H | >1 | M | Surviving and breeding without problems. Juveniles reach adulthood and multiple lab generations have been raised thus far. |
| *Asellus aquaticus* (Linnaeus, 1758) | Lummelunda, Gottland (SE) | Hand sampling (turkey baster); aquatic D-net | aquatic / surface stream | freshwater / 12 | LD/DD | H | >1 | M | Surviving and breeding without problems. Juveniles reach adulthood and multiple lab generations have been raised thus far. |
| *Asellus aquaticus* (Linnaeus, 1758) | Sušik ponor, Drežnica, Brinje (CRO) | Hand sampling (turkey baster) | aquatic / cave | freshwater / 12 | LD/DD | H | <1 | M | Surviving and breeding without problems. Juveniles reach adulthood and multiple lab generations have been raised thus far. |
| *Asellus aquaticus* (Linnaeus, 1758) | Lummelundagrottan, Gottland (SE) | Hand sampling (turkey baster) | aquatic / cave | freshwater / 12 | DD | M | <1 | L | Surviving and breeding without problems but rate of reproduction, survival of juveniles and growth rate was not sufficient for establishment of a self-sustaining colony |
| *Asellus aquaticus* (Linnaeus, 1758) | Planinska jama, Postojna (SI) | Hand sampling (turkey baster); aquatic D-net | aquatic / cave | freshwater / 12 | DD | L | NA | NA | Survived up to two months, then started dying off. No reproduction |
| *Proasellus coxalis* (Dollfus, 1892) s.l. | Lateralni kanal Vranskog jezera, Zadar (CRO) | Aquatic D-net | aquatic / surface stream | freshwater / 15 | LD/DD/WL | H | >1 | M | Surviving and breeding without problems. Juveniles reach adulthood and multiple lab generations have been raised thus far. |
| *Proasellus karamani* (Remy, 1934) | Ključka rijeka, Cerničko polje, Gacko (B&H) | Aquatic D-net | aquatic / surface stream | freshwater / 12-15 | LD/DD/WL | H | >1 | M | Surviving and breeding without problems. Juveniles reach adulthood and multiple lab generations have been raised thus far. |
| *Proasellus karamani* (Remy, 1934) | Ljuta, Konavle, Dubrovnik (CRO) | Aquatic D-net | aquatic / surface stream | freshwater / 15 | LD/DD/WL | L | NA | NA | Survived up to several months then started dying off. No reproduction. |
| *Proasellus anophtalmus* (Karaman, 1934) | Špilja kod mlina na Miljacki, Oklaj, Knin (CRO) | Hand sampling (tweezers, turkey baster) | aquatic / cave | freshwater / 12-15 | LD/DD/WL | H | <1 | M | Surviving and breeding without problems but rate of reproduction, survival of juveniles and growth rate was not sufficient for establishment of a self-sustaining colony. |
| *Proasellus anophtalmus* (Karaman, 1934) | Močiljska špilja, Dubrovnik (CRO) | Hand sampling (large pipette) | aquatic / cave | freshwater / 12-15 | LD/DD/WL | H | <1 | M | Surviving and breeding without problems but rate of reproduction, survival of juveniles and growth rate was not sufficient for establishment of a self-sustaining colony. |
| *Proasellus hercegovinensis* (Karaman, 1933) | Bjelušica, Zavala (B&H) | Hand sampling (turkey baster) | aquatic / cave | freshwater / 12 | WL | H | <1 | L | Individuals from the wild survive with no problems for a long time. We saw breeding occasionally but rate of reproduction, survival of juveniles and growth rate was not sufficient for establishment of a self-sustaining colony. |
| Sphaeromatidae |  |  |  |  |  |  |  |  |  |
| *Monolistra pretneri* (Sket, 1964) | Špilja kod mlina na Miljacki, Oklaj, Knin (CRO) | Hand sampling (turkey baster) | aquatic / cave | freshwater / 12 | WL | M | NA | NA | Survived for more than one year in the laboratory. No reproduction. |
| *Monolistra radjai* Prevorčnik & Sket, 2007 | Bunar u Raslini, Raslina, Šibenik (CRO) | Hand sampling (turkey baster) | aquatic / cave | freshwater / 12 | WL | L | NA | NA | Survived for more than one year in the laboratory. Some batches collected in the wild wouldn’t survive for long in the lab. No reproduction. |
| *Monolistra velkovrhi* (Sket, 1960) | Jastrebica, Frketić selo, Karlovac (CRO) | Hand sampling (turkey baster) | aquatic / cave | freshwater / 12 | WL | M | NA | NA | Survived for more than one year in the laboratory. No reproduction. |
| *Lekanesphaera hookeri* (Leach, 1814) | Crna Rika, Ploče (CRO) | Aquatic D-net | marine / estuary | seawater / 15 | LD/WL | M | <1 | L | Surviving up to 1 year and breeding occasionally (we observed that starvation and stress triggers mating) but juveniles don’t survive until adulthood. |
| Atyidae |  |  |  |  |  |  |  |  |  |
| *Troglocaris* sp. | Špilja pod Krogom, Mlini, Buzet (CRO) | Hand sampling (aquarium net) | aquatic / cave | freshwater / 12 | WL | H | NA | NA | Survived up to six months in the laboratory. No reproduction. |
| *Atyaephyra* sp. | Raša river, Barban (CRO) | Aquatic D-net | aquatic / surface stream | freshwater / 12 | WL | M | NA | NA | Survived up to three months in the laboratory. No reproduction. |
| Physidae |  |  |  |  |  |  |  |  |  |
| *Physella* sp. | Fogelpole Cave, Monroe County, IL (USA) | Hand sampling (tweezers) | aquatic / cave | freshwater / 20 - 22 | EE | H | > 1 | M | Surviving and breeding without problems. A hermaphroditic snail that will self-fertilize. Juveniles reach adulthood and multiple lab generations have been raised thus far. |
| *Physella* sp. | Illinois Caverns, Monroe County, IL (USA) | Hand sampling (tweezers) | aquatic/ cave | freshwater / 20 - 22 | EE | H | occasional | M | Surviving with limited reproduction in the Weck lab. |
| Physid snail | Jarun lake, Zagreb, Croatia | Hand sampling (tweezers) | aquatic/ lake | freshwater / 20 - 22 | EE | H | occasional | M | Surviving in the lab. Occasionally reproducing. |
| Planorbidae |  |  |  |  |  |  |  |  |  |
| *Ancylus* sp. | Planinska jama, Postojna (SI) | Hand sampling (tweezers) | aquatic / cave | freshwater / 12 - 15 | WL | L | NA | NA | Low survival rates, survived up to two months in the laboratory. No reproduction. |
| *Ancylus* sp. | Unica (SI) | Hand sampling (tweezers) | aquatic / surface stream | freshwater / 12-15 | LD/WL | L | NA | NA | Low survival rates, survived up to two months in the laboratory. No reproduction. |
| *Ancylus* sp. | Sušik ponor, Drežnica, Brinje (CRO) | Hand sampling (tweezers) | aquatic / cave | freshwater / 12 | WL | L | NA | NA | Low survival rates, survived up to two months in the laboratory. No reproduction. |
| *Ancylus* sp. | Sušik, Drežnica, Brinje (CRO) | Hand sampling (tweezers) | aquatic / surface stream | freshwater / 12-15 | LD/WL | M | occasional | L | Medium survival rates, survived up to six months in the laboratory and gave occasional offspring. They were kept in container with stones from natural habitat overgrown with algae. |
| Dreissenidae |  |  |  |  |  |  |  |  |  |
| *Dreissena polymorpha* (Pallas, 1771) | Jarun, Zagreb (CRO) | Hand collecting | aquatic / lake | freshwater / 12 | LD/DD/WL | H | NA | NA | Survived up to two years, then starts dying off. No reproduction. |
| *Congeria jalzici* Morton & Bilandžija, 2023 | Markov ponor, Kosinj (CRO) | Hand collecting | aquatic / cave | freshwater /12 | WL | L | NA | NA | Survived up to 6 months then starts dying off from severe fungal infection. No reproduction. |
| *Congeria kusceri* Bole, 1962 | Pukotina u tunelu polje Jezero-Peračko blato, Ploče (CRO) | Hand collecting, Sket bottle | aquatic / cave | freshwater / 12 | WL | L | NA | NA | Survived up to 6 months then starts dying off from severe fungal infection. No reproduction. |
